# Bacteriostatic Effect of a Calcined Waste Clamshell-Activated Plastic Film for Food Packaging

**DOI:** 10.3390/ma11081370

**Published:** 2018-08-07

**Authors:** Chien-Ya Kao, Yen-Chieh Huang, Sheng-Yi Chiu, Ko-Liang Kuo, Pai-An Hwang

**Affiliations:** 1 Agricultural Technology Research Institute, Hsinchu 30093, Taiwan; 9629505.bce96g@g2.nctu.edu.tw; 2 Department of Bioscience and Biotechnology, National Taiwan Ocean University, Keelung 20224, Taiwan; ja1592ck@gmail.com; 3 Water Technology Division, Material and Chemical Research Laboratories, Industrial Technology Research Institute, Hsinchu 31057, Taiwan; sy1012@itri.org.tw; 4 Seafood Technology Division, Fisheries Research Institute, Council of Agriculture, Keelung 20246, Taiwan; klkuo@mail.tfrin.gov.tw

**Keywords:** biological waste treatment, calcined waste clamshells, antibacterial, bacteriostatic effect

## Abstract

The addition of calcined waste clamshells (CCS) into polyethylene (PE) plastic bags imparts antibacterial properties due to the presence of CaO. In this study, different proportions of calcined waste clamshells were added to PE to explore its bacteriostatic effects. The PE plastic bags with 9% and 11% of CCS exhibited antibacterial efficacy. Further, total aerobic viable count (TVC) values for raw fish fillet packaging in 9% and 11% CCS-PE plastic bags for five days were similar to the 0% CCS-PE plastic bag group after three days of incubation. In addition, the CCS-PE plastic bag demonstrated stability against solvents when examined using the metal migration test under heptane, ethanol, and acetic acid treatments. The results revealed that the CCS-PE bag retains its CaO bacteriostatic efficacy and that the addition of CCS powder to PE prolongs the shelf life of raw fish fillets, as well as mitigating safety concerns from metal leakage.

## 1. Introduction

The freshwater clam, *Corbicula fluminea,* has been successfully cultured in Taiwan and the hot-water extraction of clams has been commercialized for health products [1], domestic consumption, and export to other Asian countries. Small portions of the clamshell are used as food additive and the large remainder is considered commercial waste. Over the last ten years, the fishery production of *C. fluminea* averaged more than 11,000 tons per year [2]. After removal of the edible portion (20–30% of total weight), there is approximately 8000 tons of clamshell waste produced each year. Over recent years calcined shell waste has become the focus of considerable research due to their low cost, easy accessibility, biocompatibility, and potential for chemical surface modifications using different functional groups which possess unique antibacterial activities. It is highly desirable to convert these residues into high value-added products, both from an economic and an environmental sustainability perspective, and thus research for potential uses of waste shells has been ongoing for decades. The major component of clamshell waste is calcium carbonate (CaCO_3_) which decomposes into calcium oxide (CaO) when calcined. These ingredients can be used as water treatment, and building materials, and as a catalyst for transesterification [3,4,5,6,7,8].

However, Fang et al. [9] mentions that the concentration of heavy metals in oysters are higher than that in mussels. In addition, Al-Jaberi [10] found a significant increase in the value of heavy metals (Ba, Zn, Pb, Ni, Co, Cr, Sr, Cu, Mn, and Fe) in the *C. fluminea* shell. This was really an important comment for this research, in that we needed to pay more attention to the heavy metal content, which must comply with the Taiwan Food and Drug Administration (TFDA) standard. TFDA prohibits the intentional introduction of Pb and Cd into packaging materials when selecting shell powder from different sources. Therefore, in this study, we monitored both the heavy metal content of the shell powder and the packaging materials migration test. The content of heavy metals (Pb and Cd) is less than 100 ppm by weight of the material, and heavy metals should be less than 1 ppm in the eluent of the migration test. In addition, recent publications have shown that calcined clamshells possess antibacterial properties [11,12,13]. The active oxygen generated from calcined shell powder destroys microbial cells [12]. However, in the consumer market, calcined clamshell powder is rarely sold as a household antibacterial agent. The major component of the calcined shell, CaO is a solid that has a high affinity for water which reacts with ambient moisture to form calcium hydroxide [Ca(OH)_2_]. The reaction is highly exothermic. It is also corrosive and causes significant skin, and respiratory tract irritation [14]. Therefore, it is advisable to melt-mix a polymer with calcined shell powder to prepare a polymeric antibacterial substance to maintain antibacterial properties, and minimize any adverse effects from direct contact with calcined shell powder or CaO.

Besides, antimicrobial additives are one of the food storage concepts that have been introduced to meet the consumer demand for high quality and high safety goods, with an extended shelf life. Direct addition of antimicrobials into food may result in unintended cross-reactions with food components such as lipids or proteins [15]. A possible solution is to embed antimicrobial additives into the food packaging during the manufacturing process. The use of packaging films containing antimicrobial agents could be more efficacious by controlling the migration of the antimicrobial agents into the food during the distribution process [16]. Therefore, the incorporation of antimicrobial compounds into food packaging materials has attracted considerable attention. Currently, nanostructured [17], and plant extract antimicrobial agents [18], enzymes [19], and bacteriocin [20] are commonly under investigation for use in antimicrobial packaging. Natural antimicrobials including GRAS (generally recognized as safe) and non-GRAS antimicrobials have been incorporated in plastics and tested against a variety of microorganisms [21]. However, safety and hygiene regulations, and manufacturing costs have limited the number of commercially viable and available products. Therefore, the development of an environmentally friendly and inexpensive antibacterial additive to be used in food packaging remains a matter of paramount scientific and commercial importance.

Hamester et al. [22] reported that calcined oyster and mussel shells (containing 98.2% and 95.7% CaO) have potential to replace commercial products for use as filler in polypropylene (PP). Further, 10% CaO loading in PP has no significant change in the mechanical properties of packaging. However, to our knowledge, there has been no previous work investigating the preparation of calcined waste clamshell, melt-mixed with polyethylene (PE) to be used as active packaging. Therefore, in this study, calcined waste clamshells (CCS) of various concentrations were incorporated into PE, and the CCS containing PE plastic films were characterized and analyzed by scanning electron microscope (SEM), Fourier transform infrared spectroscopy (FTIR), metal migration, and antimicrobial activity assays. The intention was to develop a material for food packaging applications.

## 2. Materials and Methods

### 2.1. Preparation of Calcined Waste Clamshell (CCS) and CCS-PE Plastic Bag

Fresh water clam (*C. fluminea*) shell waste was supplied by a restaurant in Hualien County, Taiwan. After cleaning, the clamshells were soaked in acetic acid solution, to remove the outermost stratum corneum layer and then washed with deionized water until neutralized. Shells were oven baked at 200 °C for 1 h to evaporate shell moisture and increase brittleness. The dried shell was ground to a powder using a mini-blender, then heated to 800 °C for 1.5 h, then further ground using a ball-mill without water to produce the CCS sample. The type of PE was linear low density polyethylene (LLDPE, EQUATE EFDC-7050, Shanp Deng Enterprise Co., Ltd., New Taipei City, Taiwan). The LLDPE has good toughness and puncture resistance for food packaging. The PE plastic bags and CCS containing PE (CCS-PE) plastic bags were collaboratively manufactured (Tong Yuan Plastics Co., Ltd., Chiayi County, Taiwan). Before mixing, the PE and CCS were dried in an oven. Then CCS and PE were mixed to achieve CCS concentrations of 0%, 7%, 9%. and 11% (weight percent). The mixture was then run through an injection molding machine to produce CCS-PE plastic bags measuring 20 cm × 40 cm × 0.05 mm. The extrusion temperatures and screw rotation rate were respectively 210 °C and 100 m/min. All other chemicals used were analytical grade.

### 2.2. Characterizations of CCS and CCS-PE Plastic Bags

The average particle size of CCS was determined using a Zeta sizer ZS (Malvern Instruments, Malvern, UK). The atomic composition and morphology of CCS and the CCS-PE plastic bags were determined by energy-dispersive spectrometry (EDS) coupled with SEM imaging, performed on a JEOL JSM-6500F (JEOL Ltd, Tokyo, Japan) Microscope operating at an acceleration voltage of 10 kV. The samples were mounted onto a specimen holder using a conductive adhesive. The sample was adsorbed onto Copper Conductive Tape. After any excess sample was removed, the specimen holder was dried in a contamination-free environment and stored in an electronic dry cabinet for SEM analysis. The molecular structures of CCS and CCS-PE plastic bags were investigated using FTIR-MIDAC 2000 (MIDAC Corporation, Costa Mesa CA, USA) with KBr powder, while the FTIR spectrum was recorded at wavelengths of 500–3600 nm.

### 2.3. Metal Migration Test of the CCS-PE Plastic Bag against Different Solvents

This procedure was based on the Taiwan Food and Drug Administration (TFDA) protocol for assessing material transfer from packaging materials to food [23]. Migration of materials from CCS-PE plastic bag into aqueous and fatty food simulants was carried out by total immersion of the bags in solvents, which included DD water, 4% acetic acid, 20% ethanol, and hexane. The 0%, 7%, 9%, and 11% CCS-PE plastic bags were cut into 1 cm^2^ sized film, and then the film was completely immersed in 2 mL of solution in a 10 mL tube fitted with a ground-glass stopper. Films immersed in DD water, 4% acetic acid, and 20% ethanol were stored in a controlled-temperature water bath at 60 °C and 95 °C for 30 min, those in hexane were stored at 25 °C for 60 min. After this, the samples were analyzed by inductively coupled plasma mass spectrometer (ICP-MS).

### 2.4. Digestion and ICP-MS Analysis

CCS, PE plastic bags, and migration testing samples were then analyzed for the presence of Ca, Pb, Cu, and Cd content using ICP-MS (Thermo Fisher Scientific iCAP Q_C_, Waltham MA, USA). CCS and the PE plastic bags were ground uniformly into a powder using a blender and 60 mg of dry sample was digested with 4 mL HNO_3_ using a microwave oven (Milestone ETHOS UP, Sorisole BG, Italy) for 25 min [24]. One ml of the migration testing sample was digested with 4 mL HNO_3_ under the same conditions. The operating conditions were as follows: nebulizer gas (argon) flow rate: 1.05 L/min; auxiliary gas (argon) flow: 0.8 L/min; plasma (argon) gas flow: 14 L/min; reaction gas flow (helium): 4.3 mL/min; lens voltage: 7.25 V; ICP RF power: 1550 W; CeO/Ce = 2%; and Ba^2+^/Ba = 2%. The metal isotopes used for measurements and calculations were ^44^Ca, ^208^Pb, ^63^Cu, and ^112^Cd. To check for contamination of the digestion procedure and sample manipulation, a blank solution was prepared and carried through each set of analyses. Each sample was analyzed three times and the results are expressed as mean ± SD (SD: standard deviation).

### 2.5. Antibacterial Activity of The CCS-PE Plastic Bag

This procedure was based on the Japanese Industrial Standard (JIS) Z 2801:2000 antimicrobial products test method [25]. The method had been optimized for standardizing and evaluating antimicrobial efficacy in antimicrobial products. The sample used to cover the inoculum was 5 cm × 5 cm × 0.05 mm plastic pieces cut from a sterile CCS-PE plastic bag, which had been cleaned before testing with reagent grade ethanol. The cleaned samples were air-dried for 72 h before being tested. The inoculum was prepared using *Staphylococcus aureus* ATCC 6538P and *Escherichia coli* ATCC 8739. The inoculum was diluted by nutrient broth to a target starting concentration of 5.03–5.12 Log CFU/mL. An amount of 0.4 mL of the inoculum was added to each sample, and then incubated for 24 h at 35 °C and a relative humidity of at least 90%. The inoculum in each sample was tested in triplicate at 0 and 24 h to establish organism viability. The inoculum from the sample was diluted serially by neutralizing broth and plate. All plates were incubated at 35 °C for 24 h. After incubation, bacterial colonies were counted and recorded. The antimicrobial activity = (Log CFU from 0% CCS-PE at 24 h) − (Log CFU from 7%, 9%, and 11% CCS-PE at 24 h). An antibacterial product was determined to have antibacterial effectiveness when the antibacterial activity (R) was 2.0 or more.

### 2.6. Microbial Stability of the CCS-PE Plastic Bag When Storing Raw Fish Fillets

A panel of fresh fish fillets (Tilapia, *Oreochromis mossambicus*) with unknown levels of microbial contamination was obtained from a traditional market in Keelung country, Taiwan. The 0%, 7%, 9% and 11% CCS-PE plastic bags of 20 cm × 40 cm × 0.05 mm size were cleaned with reagent grade ethanol and air-dried for 72 h before testing. The samples used to cover the inoculum were 5 cm × 5 cm × 0.05 mm plastic pieces cut from a sterile CCS-PE plastic bag, and the samples were cleaned before testing with reagent grade ethanol, then air-dried for 72 h before testing. A 10 g size raw fish fillet was placed in each CCS-PE plastic bag and packaged under degassing conditions (UV-250A, A&K Union Industrial Co., Ltd., Taichung, Taiwan). The packaged fish were refrigerated at 4 °C, then the total aerobic viable counts (TVC) were measured [26] for 5 consecutive days.

### 2.7. Statistical Analysis

Numerical data are presented as means ± standard deviation. The data was analyzed by a one-way analysis of variance (ANOVA) followed by the Least Significant Difference test using SPSS (Chicago, IL, USA) version 10 software. *p* < 0.05 was considered a significant difference.

## 3. Results

### 3.1. Morphological Observations of the CCS Powder and CCS-PE Plastic Bag

**Figure 1:**
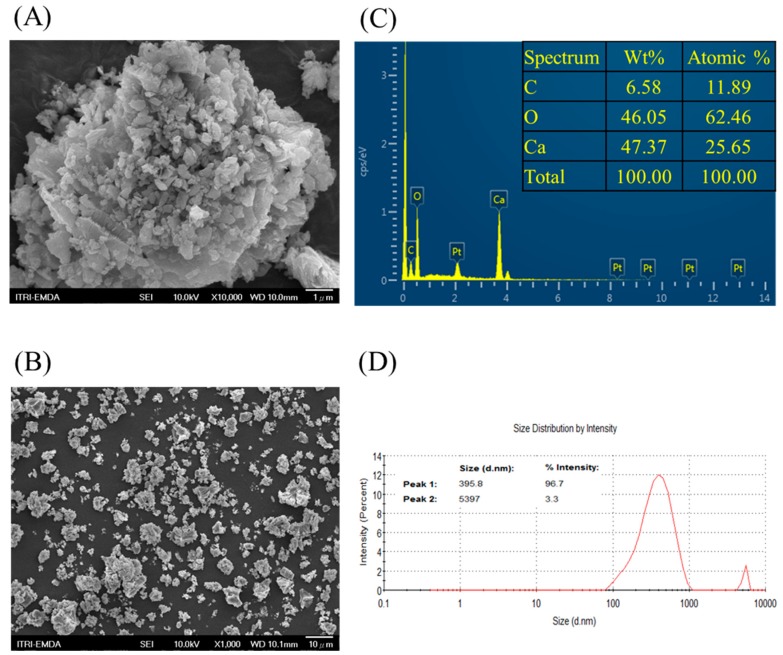
Scanning electron microscope (SEM) image of calcined waste mussel shell (CCS) at 10,000× (**A**) and 1000× (**B**) magnification, as well as energy-dispersive spectroscopy (EDS) spectrum (**C**) and particle size distribution (**D**) of CCS.

The PE plastic antimicrobial additive, CCS that was used in the present study was derived from *C. fluminea* shells calcined at 800 °C for 1.5 h. Hu et al. [27] reported that the calcination temperature is over 800 °C, the diffraction patterns of calcined freshwater clamshells are characteristic of CaO, caused by the decomposition of CaCO_3_ from the natural shell. The morphology of CCS powder was examined by SEM and representative images of CCS powder are shown in Figure 1A,B at a magnification of 10,000× and 1000×. The CCS powder aggregated together and presented a porous structure. Local chemical analysis by EDS indicated that the major elements were Ca (47.37% weight and 25.65% atomic weight), O (46.05% weight and 62.46% atomic weight) and C (6.58% weight and 11.89% atomic weight) for CCS powder (Figure 1C), with an average particle diameter of approximately 395.8 nm (96.7%) (Figure 1D), which was consistent with the measurement from dynamic light scattering.

**Figure 2:**
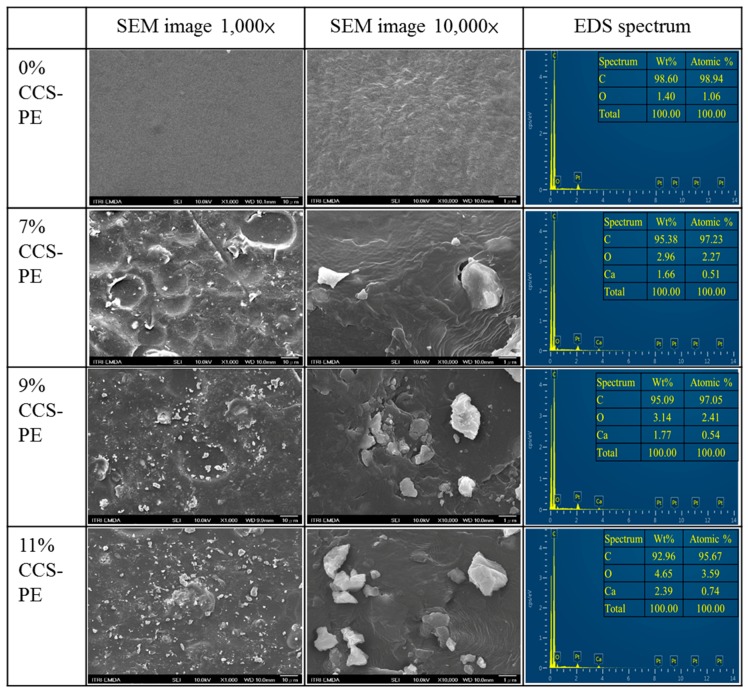
Scanning electron microscope (SEM) image of 0%, 7%, 9%, and 11% CCS-PE plastic bags at 1000×, 10,000× magnification, and the corresponding energy-dispersive spectroscopy (EDS) spectrum.

The surface morphology of the CCS-PE plastic bag was also examined by SEM, and representative images of samples with 7%, 9%, and 11% CCS are shown in Figure 2. The white dots in the micrographs correspond to CCS that exhibited irregular particle shape. Under a magnification of 1,000×, the 9% and 11% CCS bags with higher loading displayed well-dispersed CCS, but still had a few small CCS clusters. On the other hand, the 7% CCS-PE plastic bag had a rough morphology, while the 9% and 11% CCS-PE plastic bags were considerably smoother, indicating CCS powder present in the PE plastic bag. Díez-Pascual and Díez-Vicente [28] also reported that 10% ZnO-poly (3-hydroxybutyrate) (PHB) film has smoother morphology than 1% ZnO-PHB film. In addition, Yao et al. [29] and Shnawa et al. [30] indicated that the crystallinity and thermal stability of PE increases with increasing clamshell content, while clamshell content can promote the heterogeneous nucleation of PE to make a different surface morphology. When we observed the CCS-PE plastic bag at a magnification of 10,000×, we observed that CCS was embedded in the PE film, with partial CCS particles stuck in the PE film and partial CCS particles exposed. Therefore, some CCS might be in direct contact with the contents of the PE plastic bag, thus we performed a migration test with the CCS-PE plastic bag under different solvents. The atomic composition of the CCS-PE plastic bags was also determined by SEM–EDS, and it was confirmed that Ca increased as more CCS was added (Figure 2).

### 3.2. FTIR Spectra of CCS Powder and CCS-PE Plastic Bags

**Figure 3:**
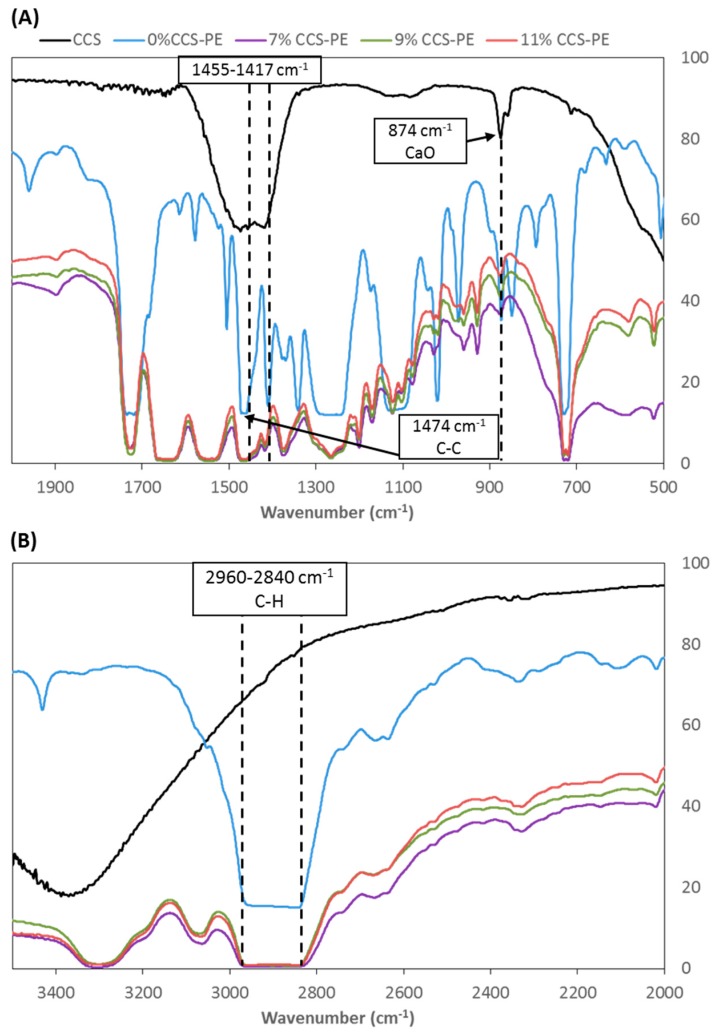
Fourier transform infrared (FTIR) spectra of CCS powder, 0%, 7%, 9%, and 11% CCS-PE plastic bags of (**A**) 500–2000 cm^−1^ and (**B**) 2000–3500 cm^−1^.

FTIR spectra analysis provided further evidence that CCS powder was present in the PE plastic bag. The FTIR spectra of CCS at 0%, 7%, 9%, and 11% of the CCS-PE plastic bags are shown in Figure 3. CCS was mainly active in two bands, 874 cm^−1^ and 1417–1455 cm^−1^ (Figure 3A). The appearance of strong IR absorption band at 874 cm^−1^ was attributed to CaO [31], and the bands between 1417 and 1455 cm^−1^ corresponded to the symmetric stretching vibration of unidentate carbonate. This was due to the exposure of the highly reactive CaO surface area to air during calcination, resulting in the formation of large amounts of CO_2_ and H_2_O, which are adsorbed on the surface of CaO in the form of free –OH and carbonate [32]. Perea et al. [33] also show that calcined shell has a peak around 1450 cm^−1^, and our results agreed with their findings. On the other hand, pure PE plastic bags (0% CCS-PE) displayed a very strong band between 2840 and 2960 cm^−1^ (Figure 3B) and another band at 1474 cm^−1^. The band in the range of 2840 and 2960 cm^−1^ corresponds to C–H stretching vibration and the band at 1474 cm^−1^ corresponds to a C–C stretching bond, which matches the characteristic PE absorbance bands [34]. In addition, there is a broad band between 3300–3400 cm^−1^, this can be attributed to the stretching and bending vibrations of hydrogen-bonded surface OH groups of the physisorbed water. This reveals that water molecules are retained in the fabricated CaO and CaCO_3_ from the CCS [35]. The spectra of the 7%, 9%, and 11% CCS-PE plastic bags had characteristic bonds of both CCS and pure PE plastic bags. Further, the bond related to CaO stretching at 874 cm^−1^ was more intense, in agreement with its higher CCS loading.

### 3.3. Migration Test of the CCS-PE Plastic Bag under Different Solvents

The morphological observation of SEM imaging at a magnification of 10,000×, showed that CCS was present in the PE film, some CCS partially exposed. Thus, some CCS might be in direct contact with the contents of the package. This raises fears of possible metal leakage into the contents of the package. We investigated the likelihood of metal contamination by performing a migration test of the CCS-PE plastic bag under different solvents. It has been acknowledged that some compounds present in plastic food containers can migrate into food [36,37]. The TFDA prohibits the intentional introduction of Pb and Cd into packaging materials. It also sets a limit on the presence of these regulated heavy metals (Pb and Cd) to below 100 ppm by weight of the material, and that heavy metals should be below 1 ppm in the eluent of the migration test [23]. Due to industrialization, pollutants have caused biogeochemical changes which might be a source of heavy metals in river water bodies, and these heavy metals affect the growth of the shell and can accumulate in it [38]. Al-Jaberi [10] reports that the content of heavy metals in *C. fluminea* shells has increased significantly. According to the results of Figure 2, CCS made from the *C. fluminea* shell was embedded into PE film, and might be in direct contact with food. Here the four atoms quantified using ICP-AES were Ca, Pb, Cu, and Cd.

The major composition of CCS was 53.1% Ca, with trace Pb (2.58 ± 0.07 μg/g), Cu (14.50 ± 0.13 μg/g) and Cd (17.42 ± 0.31 μg/g). The PE plastic bag (0% CCS-PE) also had trace Ca (456.2 ± 3.14 μg/g), Pb (53.10 ± 0.12 μg/g), Cu (15.36 ± 0.55 μg/g), and Cd (32.65 ± 0.06 μg/g). The Pb and Cd contents of the CCS and PE plastic bags were under 100 ppm and thus, in compliance with regulations (Table 1). Results for Ca, Pb, Cu, and Cd migration from 0%, 7%, 9%, and 11% CCS-PE plastic bags are shown in Table 1. Pb and Cd were not detected in all samples from the migration test, only Cu showed trace migration. The Pb and Cd contents of the migration samples from 0%, 7%, 9%, and 11% CCS-PE plastic bags were in compliance with the regulation of under 1 ppm. Interestingly, the amount of Ca was high in all samples from the migration test, and Ca content was higher in the 4% acetic acid migration sample than in the DD water, 20% ethanol and heptane migration samples. In a 4% acetic acid solution Ca content was higher at 95 °C than at 60 °C. The observation suggests that exposure to higher temperatures while heating food, might result in an increase of Ca migration from the CCS-PE plastic bag to food. Loyo-Rosales et al. [39] also reported that high temperature accelerates the migration of substances from a plastic container onto food. In addition, it has been shown that larger particle sized additives for plastic have a higher migration [40]. The CCS particle size was around 395.8 nm (Figure 1D), which is considered a large sized additive for plastic, and may explain the high Ca migration of the CCS-PE plastic bag. According to the above results, the amount of Ca was high in all samples in the migration test, especially in the migration sample of 4% acetic acid solvent at 95 °C for 30 min, however, the pH values did not change significantly in all samples. Whether or not the CCS-PE plastic bag was suitable for long-term packaging still required further testing.

### 3.4. Antimicrobial Efficacy of the CCS-PE Plastic Bag against *Staphylococcus aureus* ATCC 6538P and Escherichia coli ATCC 8739

Recent publications have shown that calcined clamshells possess antibacterial properties [11,12,13]. The active oxygen generated from calcined shell powder destroys microbial cells [12]. The incorporation of antimicrobial compounds into food packaging materials has attracted considerable attention. In this regard, the antibacterial action of 0%, 7%, 9%, and 11% CCS-PE plastic bags was tested against two human pathogen bacteria: *S. aureus* (Gram-positive) and *E. coli* (Gram-negative), and the results are shown in Table 2. After incubation, bacterial colonies decreased exponentially with increasing CCS concentration. According to the Japanese Industrial Standard (JIS) Z 2801:2000 antimicrobial products test method [25], an antibacterial product is determined to have antibacterial efficacy when the antibacterial activity (R) is 2.0 or more. We observed that the antibacterial activity (R) values of 9% and 11% CCS-PE plastic bags were over or very near 2.0. The larger CCS exposed surface area might explain the stronger antibacterial efficacy of 9% and 11% CCS-PE plastic bags. That bactericidal effects were from the alkaline effects of CaO in the bag [41]. CaO is the major component of the calcined shell. It generates reactive oxygen, such as superoxide anions, and this activity has been observed in the powder slurry [42]. The changes in antibacterial sensitivity of *E. coli* and *S. aureus* when treated with calcined marine shells are consistent with the changes caused by reactive oxygen treatment. It has been suggested that reactive oxygen generated from CaO/calcined marine shells is also a primary mechanism of antibacterial activity [12]. CaO is a solid that has a high affinity for water and it reacts with ambient moisture to form calcium hydroxide [Ca(OH)_2_] [42]. A feasible antibacterial mechanism of CCS-PE plastic bags could be that CaO/CCS reacts with inoculum to form Ca(OH)_2_ with heat emission, and Ca(OH)_2_ alters the biologic properties of the bacterial lipopolysaccharides in the cell walls and inactivates membrane transport mechanisms, resulting in cell toxicity [43]. The primary advantage of Ca(OH)_2_ is its ability to kill microorganisms without direct contact, by absorbing the CO_2_ required for bacterial growth [44].

Furthermore, the antibacterial efficacy of CCS-PE plastic bags against *E. coli* was stronger than *S. aureus*. The different behavior observed against *E. coli* and *S. aureus* was attributed to compositional differences of the cell surface [45]. Sawai et al. [12] also reported that the *S. aureus* strain is more resistant to calcined shell powder treatment than *E. coli*, *Salmonella typhimurium,* and *Bacillus subtilis*.

### 3.5. Bacteriostatic Efficacy of CCS-PE Plastic Bags on TVC of Raw Fish Fillet at 4 °C

**Figure 4:**
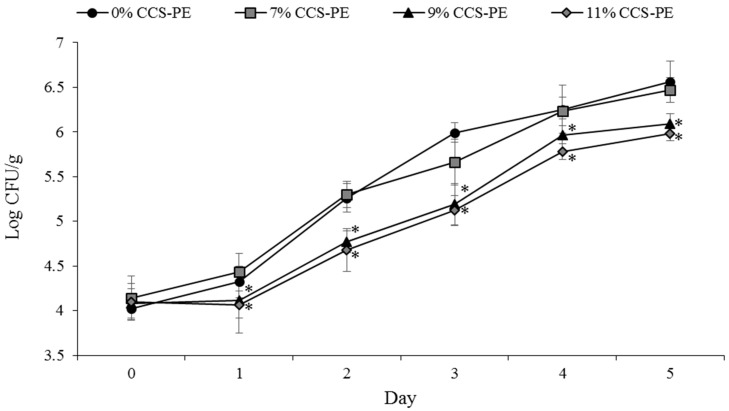
Effect of 0%, 7%, 9%, and 11% CCS-PE plastic bags on the total aerobic viable counts (TVC) of raw fish fillet. Results are reported as mean ± SD (*n* = 5) colony forming units (CFU) (Log_10_/g). * *p* < 0.05 when compared with 0% CCS-PE plastic bag group.

The above results demonstrate the potential of CCS-PE packaging in preventing bacterial contamination. The CCS exhibits antimicrobial efficacy and also reduces the risk of direct contact with stimuli by fusing with PE. Here, we investigated the bacteriostatic efficacy of the CCS-PE plastic bag by testing it with the model food system, fish fillet, at 4 °C. The mean initial microbial population immediately after packaging of the raw fish fillet was observed at 4.08 Log CFU/g. The TVC curves of raw fish fillet packaging using the 0% CCS-PE plastic bag demonstrated a relationship between microbial population and storage time. In all packages except the 0% and 7% CCS-PE plastic bag, a significant decrease in the microbial population (*p* < 0.05) was observed. By increasing the CCS concentration, the bacteriostatic efficacy of the PE plastic bag increased. The TVC values of the raw fish fillet packaged in 9% and 11% CCS-PE plastic bags for 5 days were observed at 6.09 and 5.98 Log CFU/g, which were close to the measurements of the 0% CCS-PE plastic bag group after 3 days of incubation (5.99 Log CFU/g) (Figure 4). It was suggested that 9% and 11% CCS-PE plastic bags could prolong the storage time of raw fish fillets, but 11% CCS-PE plastic bags did not show stronger bacteriostatic efficacy than the 9% CCS-PE plastic bags in our food system. Sawai et al. [46] reported that neither CaO nor calcined shell powder are mutagenic in the Ames assay, and CaO can reduce the mutagenicity of the mutagen [47]. Therefore, use of CCS as an antibacterial or bacteriostatic material has a lower safety concern. In addition, CCS could revert into CaCO_3_ by absorbing CO_2_ from air when the CCS-PE plastic degrades, which would give fewer metallic pollution concerns than other antibacterial metal oxides like MgO, ZnO, Al_2_O_3_, CuO, Cr_2_O_3_, Ni_2_O_3_ or Mn_2_O_3_ [48].

## 4. Conclusions

This study demonstrated that calcined waste clamshell powder present on PE film has bacteriostatic efficacy. The morphological observation by SEM imaging of the CCS-PE plastic bag showed that the CCS-PE plastic bags had a smooth morphology. The CCS powder and CCS-PE plastic bag were characterized by FTIR techniques and were mainly active with three bands, 874 cm^−1^, 1417–1455 cm^−1^ and 2840–2960 cm^−1^. In addition, the CCS-PE bags demonstrated antibacterial efficacy and better TVC values when used in the packaging of raw fish fillets in 9% and 11% CCS-PE plastic bags than that of 0% CCS-PE plastic bag. We thus demonstrated that the addition of CCS powder into PE could retain bacteriostatic efficacy and prolong the shelf life (2 days) of the product without concern of metal leakage. This demonstrates the potential application of using CCS-PE.

## Figures and Tables

**Table 1:** The calcium and three heavy metals in calcined waste clam shell (CCS), PE plastic bag (0% CCS-PE) and the migration test from CCS-PE plastic bags with different treatments.

Samples				Ca	Pb	Cu	Cd
				(μg/g)			
CCS				53.1%	2.58 ± 0.07	14.50 ± 0.13	17.42 ± 0.31
PE plastic bag				456.2 ± 3.14	53.10 ± 0.12	15.36 ± 0.55	32.65 ± 0.06
**Migration test**	**Solvent**	**Treatment**		**Ca**	**Pb**	**Cu**	**Cd**
		**Temperature**	**Time**	**(ppb)**			
0% CCS-PE	DD water	60 °C	30 min	10.92 ± 0.23	nd	nd	nd
		95 °C	30 min	18.82 ± 0.17	nd	nd	nd
	4% acetic acid	60 °C	30 min	15.75 ± 0.36	nd	nd	nd
		95 °C	30 min	21.14 ± 0.22	nd	nd	nd
	20% ethanol	60 °C	30 min	16.83 ± 0.29	nd	nd	nd
		95 °C	30 min	15.54 ± 0.16	nd	nd	nd
	heptane	25 °C	60 min	19.24 ± 0.15	nd	nd	nd
7% CCS-PE	DD water	60 °C	30 min	106.33 ± 0.30	nd	nd	nd
		95 °C	30 min	167.20 ± 0.21	nd	nd	nd
	4% acetic acid	60 °C	30 min	308.82 ± 0.18	nd	nd	nd
		95 °C	30 min	551.70 ± 0.14	nd	nd	nd
	20% ethanol	60 °C	30 min	239.10 ± 0.17	nd	nd	nd
		95 °C	30 min	370.90 ± 0.36	nd	nd	nd
	heptane	25 °C	60 min	112.21 ± 0.31	nd	nd	nd
9% CCS-PE	DD water	60 °C	30 min	88.44 ± 0.12	nd	nd	nd
		95 °C	30 min	113.13 ± 0.28	nd	nd	nd
	4% acetic acid	60 °C	30 min	381.40 ± 0.15	nd	nd	nd
		95 °C	30 min	523.34 ± 0.20	nd	nd	nd
	20% ethanol	60 °C	30 min	167.44 ± 0.19	nd	nd	nd
		95 °C	30 min	200.31 ± 0.18	nd	nd	nd
	heptane	25 °C	60 min	198.16 ± 0.25	nd	nd	nd
11% CCS-PE	DD water	60 °C	30 min	138.01 ± 0.24	nd	nd	nd
		95 °C	30 min	142.01 ± 0.18	nd	nd	nd
	4% acetic acid	60 °C	30 min	711.07 ± 0.35	nd	nd	nd
		95 °C	30 min	841.22 ± 0.29	nd	nd	nd
	20% ethanol	60 °C	30 min	178.60 ± 0.20	nd	nd	nd
		95 °C	30 min	163.11 ± 0.24	nd	nd	nd
	heptane	25 °C	60 min	134.69 ± 0.15	nd	nd	nd

Data are reported as mean ± SD (*n* = 3). Nd: not detected. (All results are reported with Pb < 2 ppb, Cu < 0.5 ppb, and Cd < 1 ppb, respectively.).

**Table 2:** Effect of 0%, 7%, 9%, and 11% CCS-PE plastic bags on the antibacterial activity against *Staphylococcus aureus* ATCC 6538P and *Escherichia coli* ATCC 8739.

Samples	Log CFU/mL at 0 h	Log CFU/mL at 24 h	Antimicrobial activity (R) *
*Escherichia coli* ATCC 8739			
0% CCS-PE	5.12 ± 0.10 **	8.13 ± 0.21	
7% CCS-PE	5.12 ± 0.10	6.84 ± 0.28	1.29
9% CCS-PE	5.12 ± 0.10	5.68 ± 0.16	2.45
11% CCS-PE	5.12 ± 0.10	5.75 ± 0.08	2.38
*Staphylococcus aureus* ATCC 6538P			
0% CCS-PE	5.03 ± 0.07	7.96 ± 0.18	
7% CCS-PE	5.03 ± 0.07	7.13 ± 0.15	0.83
9% CCS-PE	5.03 ± 0.07	6.15 ± 0.25	1.81
11% CCS-PE	5.03 ± 0.07	5.60 ± 0.35	2.36

* Antimicrobial activity = (Log CFU from 0% CCS-PE at 24 h) − (Log CFU from 7%, 9% and 11% CCS-PE at 24 h), an antibacterial product was determined to have antibacterial effectiveness when the antibacterial activity (R) was 2.0 or more. ** Results are reported as mean ± SD (*n* = 5) colony forming units (CFU) (log10/mL).
